# Birth weight trends in England and Wales (1986–2012): babies are getting heavier

**DOI:** 10.1136/archdischild-2016-311790

**Published:** 2017-08-05

**Authors:** Rebecca Elisabeth Ghosh, Jacob Dag Berild, Anna Freni Sterrantino, Mireille B Toledano, Anna L Hansell

**Affiliations:** 1 UK Small Area Health Statistics Unit, MRC-PHE Centre for Environment and Health, School of Public Health, Imperial College London, London, UK; 2 Imperial College Healthcare NHS Trust, London, UK

**Keywords:** Epidemiology, Qualitative Research, Growth

## Abstract

**Introduction:**

Birth weight is a strong predictor of infant mortality, morbidity and later disease risk. Previous work from the 1980s indicated a shift in the UK towards heavier births; this descriptive analysis looks at more recent trends.

**Methods:**

Office for National Statistics (ONS) registration data on 17.2 million live, single births from 1986 to 2012 were investigated for temporal trends in mean birth weight, potential years of birth weight change and changes in the proportions of very low (<1500 g), low (<2500 g) and high (≥4000 g) birth weight. Analysis used multiple linear and logistic regression adjusted for maternal age, marital status, area-level deprivation and ethnicity. Additional analyses used the ONS NHS Numbers for Babies data set for 2006–2012, which has information on individual ethnicity and gestational age.

**Results:**

Over 27 years there was an increase in birth weight of 43 g (95% CI 42 to 44) in females and 44 g (95% CI 43 to 45) in males, driven by birth weight increases between 1986–1990 and 2007–2012. There was a concurrent decreased risk of having low birth weight but an 8% increased risk in males and 10% increased risk in females of having high birth weight. For 2006–2012 the birth weight increase was greater in preterm as compared with term births.

**Conclusions:**

Since 1986 the birth weight distribution of live, single births in England and Wales has shifted towards heavier births, partly explained by increases in maternal age and non-white ethnicity, as well as changes in deprivation levels. Other potential influences include increases in maternal obesity and reductions in smoking prevalence particularly following the introduction of legislation restricting smoking in public places in 2007.

**What is already known on this topic?**

- Birth weight is a strong predictor of infant mortality, morbidity and later disease risk.
- Work in the UK, Europe and America indicated a shift towards heavier births, it is unknown if this trend has continued in England and Wales.

**What this study adds?**

- Analysis of 17 million births using national birth statistics data 1986–2012 shows that babies in England and Wales have become heavier over the past three decades.
- We found an adjusted 4% decrease in risk of low birth weight with a corresponding 8%–10% increase risk of high birth weight.
- Increases in birth weight plateaued in 1993–2006 but increased again from 2007, coincident with the introduction of legislation banning smoking in public places.

## Introduction

Birth weight is a strong predictor of infant mortality^1^ and morbidity,^2^ with both low and high birth weight a concern to public health. Low birth weight is associated with increased mortality and morbidity in infancy as well as later life.^2 3^ A higher than average birth weight is associated with increased risks of infant mortality, adult obesity and obstetric complications such as caesarean section.^4–6^ The birth weight of a newborn will depend on the length of the pregnancy (gestation) and fetal (intrauterine) growth.^7 8^ These are influenced by clinical factors relating to the fetus or mother, as well as other maternal factors such as body mass index (BMI), age, socioeconomic status and ethnicity.^9–12^

Previous analyses indicate that the distribution of birth weight in Britain shifted towards heavier births during the 1980s^13–15^; the proportion of high birth weight babies also increased in the 1980s,^16^ levelling off in England and Wales during the 1990s, but it is unknown if these trends have continued. A similar upward trend was observed in other Western European countries such as Norway,^17^ Sweden,^18^ Denmark,^19^ France^20^ and North America,^5^ although from the 2000s this trend appeared to reverse in France, the USA and Germany.^20–22^ Relatively small increases or decreases in birth weight are likely to have a limited effect on health at an individual level, but at the population level may reflect important changes in birth weight distribution with potential impacts on risk of mortality and morbidity for births and future population health. Monitoring trends in birth weight, and the factors that affect birth weight, helps in planning and evaluating public health interventions.

Analysing live singleton births for England and Wales from 1986 to 2012, the aims of this paper are to investigate:

1. temporal trends in the mean birth weight
2. potential years of change in the mean birth weight
3. changes in proportions of births that are low or high birth weight.

## Methods

Analyses used England and Wales data from the Office for National Statistics (ONS) births data set (1986–2012) and the NHS Numbers for Babies (NN4B) data set (2006–2012) supplied by ONS. All births must be registered at a local register office and the information is compiled nationally by the General Register Office.^23^ The ONS births data set is considered to be complete and of high quality,^24^ but does not include data on gestational age and ethnicity, key predictors of birth weight, although it does include parents’ countries of birth and parental occupation. The NN4B data set has comparable completeness and quality to ONS births^25^ and has information on gestational age and babies’ ethnicity, fetalneonatal-2016-311790but is only available from 2006.^24^

Exclusions were made for stillbirths, multiple births and birth weights <500 g or >6000 g. In the NN4B data gestational ages <24 or >44 weeks were excluded due to potential inconsistencies in the recording of gestational age and birth weight.^24^ Births with missing information on birth weight, maternal age and/or postcode (preventing linkage to area-level indicators) were also excluded and compared with those included using the Χ^2^ test or t-test. From 1986 to 2012, 247 435 (1.4%) records were excluded from ONS births due to missing data (online supplementary figure 1A), and from 2006 to 2012, 77 627 (1.6%) records were excluded from NN4B data due to missing data (online supplementary figure 1B).

10.1136/archdischild-2016-311790.supp1 Supplementary file 1

An area-level indicator of socioeconomic status was used as individual-level indicators are not available for the whole data set. Each birth was assigned a Carstairs index 2001^26^ quintile based on postcode registered on the birth certificate; Carstairs 2001 was chosen as it is a score from the midpoint of the study period that covers both England and Wales. Area-level ethnicity was defined as the percentage of non-white individuals in each 2001 census output area (COA). Individual ethnicity in NN4B, based on the mother’s report of ethnicity of the baby, was defined as white, black, Asian or other ethnicity.

Low birth weight categories were based on definitions set by the WHO,^27^ while the high birth weight cut-off was chosen to be consistent with ONS statistics.^28^ The outcomes were the following:

1. yearly change in mean birth weight
2. change in birth weight from 1986 to 2016
3. proportion of infants born with a:
  - very low birth weight (VLBW) <1500 g
  - low birth weight (LBW) <2500 g
  - high birth weight (HBW) ≥4000 g
4. NN4B only (2006–2012) — all outcomes split by preterm and term (≥37 weeks gestation) births.

Temporal trends in birth weight and in birth weight groups were assessed yearly using multiple linear and logistic regression. Analyses were stratified by sex and adjusted for maternal age, marital status, area-level deprivation and ethnicity. Potential temporal points of change in the average yearly birth weight were identified with a change point analysis using binary segmentation algorithm allowing for multiple change points.^29^ Due to an error in the ONS births maternal age variable for 1991, multiple ordered logistic regression was used to impute new maternal age observations for 1991 using 1989–1990 and 1992–1993.

All analyses were performed using R V.3.2.3 and Stata V.13.0.

## Results

From 1986 to 2012, 17 254 624 live, singleton ONS births had complete data and were included. Excluded births had a 38 g lower mean birth weight and lived in more deprived areas with a higher proportion of non-white ethnic groups (online supplementary table 1). From 2006 to 2012, 4 708 769 live, singleton births were included from NN4B. Excluded NN4B births had a 137 g lower mean birth weight, were more likely to be younger mothers, lived in more deprived areas, were less likely to be white and had a lower mean gestational age (1 week) (online supplementary table 2).

**Figure 1:**
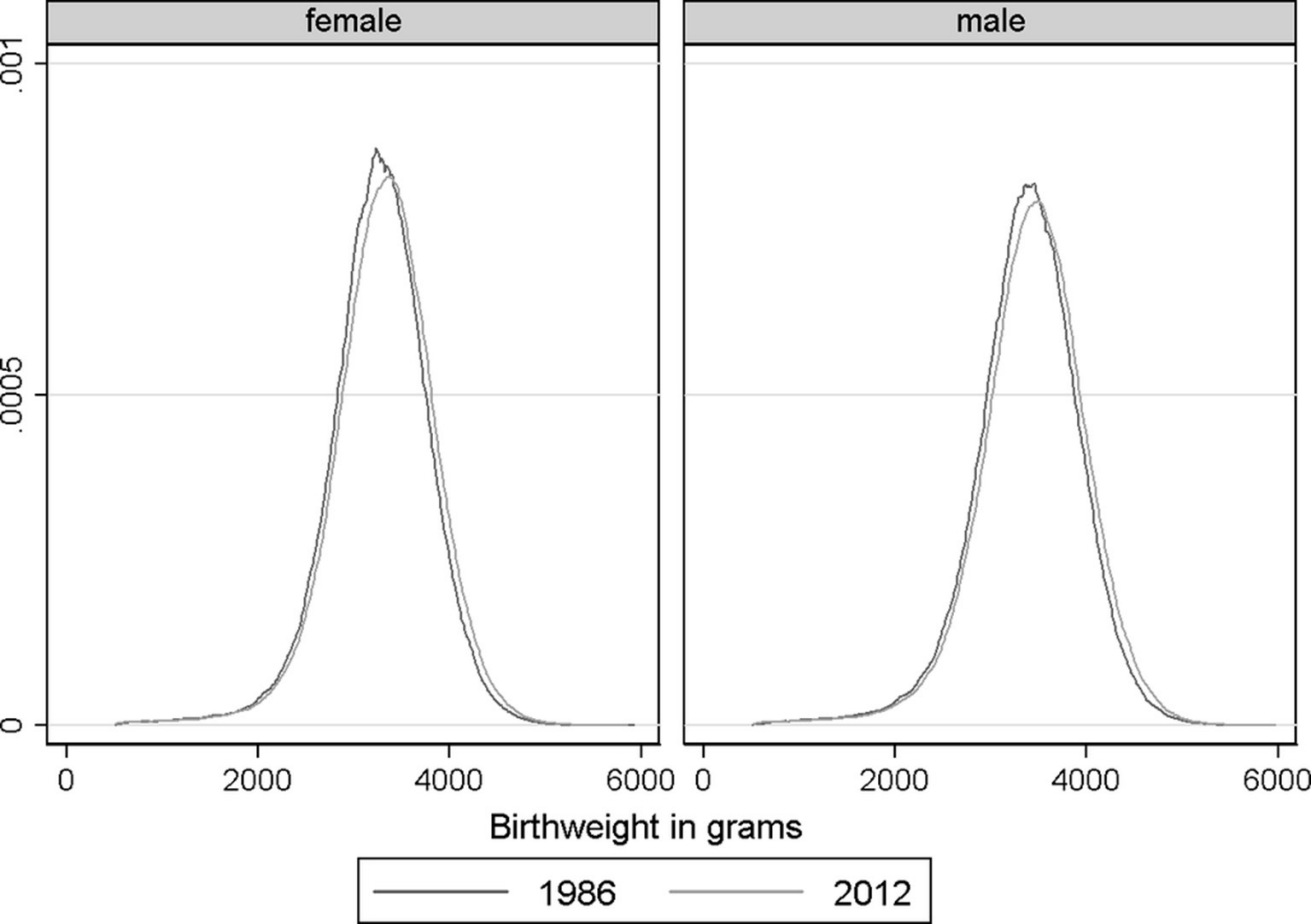
Birth weight distribution in live singletons by sex in 1986 and 2012 in England and Wales.

From 1986 to 2012, the mean birth weight increased by 58 g from 3258 g to 3316 g for females, and from 3376 g to 3436 g (60 g) for males (table 1). There was a shift in the distribution of birth weight in both females and males with a disproportionate increase in higher birth weights (figure 1). This was also seen in the changes in the 10th and 90th birth weight centiles, which showed a larger increase in the 90th centile between 1986 and 2012 (table 1).

**Table 1:** Descriptive statistics of live, singleton births in 1986 and 2012 and the difference between the years in England and Wales

	1986	2012	Difference (2012–1986)
	Female		
Live-born singletons (n)	314 100	340 490	26 390
Mean birth weight (g)	3258	3316	58
Median birth weight (g)	3280	3340	60
10th centile	2640	2680	40
90th centile	3600	3660	60
VLBW (%)	0.72	0.72	0.00
LBW (%)	6.39	5.77	−0.62
HBW (%)	6.72	8.84	2.12
	Male		
Live-born singletons (n)	330 721	359 109	28 388
Mean birth weight (g)	3376	3436	60
Median birth weight (g)	3400	3460	60
10th centile	2720	2765	45
90th centile	4030	4110	80
VLBW (%)	0.74	0.72	−0.02
LBW (%)	5.44	4.85	−0.59
HBW (%)	11.63	14.57	2.94

HBW, high birth weight; LBW, low birth weight; VLBW, very low birth weight.

**Figure 2:**
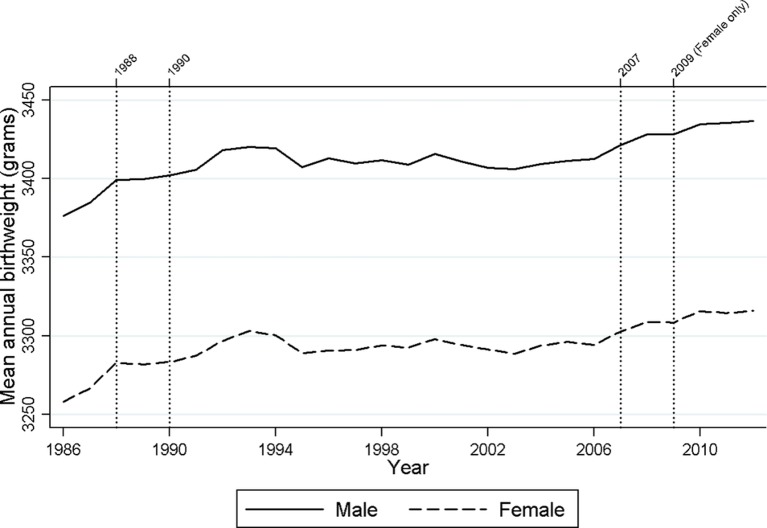
Temporal trends in the mean birth weight of all live, singleton births in England and Wales (1986–2012) with years of change.

Figure 2 shows the overall change in the annual mean birth weight for females and males, with the years of potential change indicated. Four change points were identified in the birth weight trends for females, but only three for males. The increase in birth weight appeared to be driven by larger changes between 1986 and 1990 and from 2007 onwards, with the 16-year period between showing little change in annual trends.

Table 2 presents modelled mean birth weight trends in ONS data from 1986 to 2012. In the unadjusted analysis there was a yearly increase in mean birth weight of 1.4 g for females and 1.4 g for males. After adjustment for maternal age, marital status, area-level deprivation and ethnicity, the yearly change increased to 1.6 g (95% CI 1.5 to 1.6) for females and 1.6 g (95% CI 1.6 to 1.7) for males, equivalent to a 43 g increase in female births and 44 g increase in male births between 1986 and 2012. Modelled trends for NN4B (2006–2012) using similar adjustments showed higher increases in birth weight. When stratified by term/preterm births, the increase in birth weight was greater in preterm (<37 weeks’ gestation) births (table 2). In a sensitivity analysis the risk of being born preterm showed a slight decrease in the NN4B data between 2006 and 2012 (online supplementary table 3). When stratifying by maternal age (<30 years/≥30 years), the increase in birth weight over time was greater in older mothers (online supplementary table 4).

**Table 2:** Temporal trends in mean birth weight (g) in all births 1986–2012 (ONS data) and all, term and preterm births 2006–2012 (NN4B data)

	Unadjusted yearly birth weight change (g) (95% CI)	Adjusted* yearly birth weight change (g) (95% CI)	Unadjusted birth weight change (g) (95% CI) for whole study period†	Adjusted birth weight change (g) (95% CI) for whole study period†
ONS births 1986–2012				
Female births	1.4 (1.3 to 1.4)	1.6 (1.5 to 1.6)	37.0 (35.9 to 38.3)	42.9 (41.6 to 44.0)
Male births	1.4 (1.4 to 1.5)	1.6 (1.6 to 1.7)	38.1 (36.7 to 39.2)	44.0 (42.7 to 45.4)
NN4B 2006–2012				
Female				
All births	3.4 (3.0 to 3.7)	3.9 (3.6 to 4.3)	23.7 (21.2 to 26.1)	27.6 (25.1 to 30.1)
Term births	2.3 (1.9 to 2.6)	2.7 (2.4 to 3.1)	15.8 (13.6 to 18.1)	19.2 (16.9 to 21.4)
Preterm births	4.3 (2.3 to 6.2)	4.3 (2.4 to 6.3)	29.8 (16.3 to 43.3)	30.2 (16.5 to 44.1)
Male				
All births	3.8 (3.4 to 4.1)	4.4 (4.0 to 4.8)	26.5 (23.9 to 29.0)	30.9 (28.3 to 33.5)
Term births	2.5 (2.2 to 2.8)	3.0 (2.7 to 3.3)	17.6 (15.4 to 19.9)	21 (18.8 to 23.3)
Preterm births	4.4 (2.3 to 6.2)	4.3 (2.5 to 6.1)	30.6 (16.0 to 43.1)	30.2 (17.4 to 43.0)

*Adjusted for maternal age, marital status, area-level deprivation, area-level ethnicity (ONS) and individual ethnicity (NN4B).

†Birth weight change pooled for whole study period: ONS (1986–2012), NN4B (2006–2012).

NN4B, NHS Numbers for Babies; ONS, Office for National Statistics.

The proportion of births with a very low birth weight remained stable over the study period for both sexes (online supplementary figure 2). The proportion of a low birth weight births showed an initial increase up to the early 2000s before declining. The proportion of a high birth weight births increased for females (6.7% 1986 to 8.8% 2012) and males (11.6% 1986 to 14.6% 2012).

From 1986 to 2012, there was a 1%–2% decrease in the adjusted risk of being born a very low birth weight and 4% decrease in the risk of being born a low birth weight, but an 8%–10% increased risk of being born a high birth weight (table 3). For 2006–2012 similar trends were seen but with larger changes in risk. The adjusted risk of being born a very low birth weight decreased by 9%–12%, the risk of being born a very low birth weight decreased by 6%, and there was a 2%–3% increased risk of being a high birth weight. When the analysis was split between term and preterm births, the decreased risk of being born a very low birth weight was only significant in preterm births, with a 6% decrease. The decreased risk of being a low birth weight was significant in males in both term and preterm births, but for females only in term births.

**Table 3:** Risks of being born in categories of birth weight in all births 1986–2012 (ONS data) and all, term and preterm births 2006–2012 (NN4B data)*

	Unadjusted yearly OR (95% CI)	Adjusted† yearly OR (95% CI)	Unadjusted† yearly OR (95% CI) for whole study period	Adjusted† yearly OR (95% CI) for whole study period‡
ONS births 1986–2012				
Female				
VLBW	1.00	1.00	1.00	0.99
LBW	1.00	1.00	1.00	**0.96**
HBW	**1.01**	**1.01**	**1.12**	**1.10**
Male				
VLBW	1.00	1.00	1.00	**0.98**
LBW	1.00	1.00	1.00	**0.96**
HBW	**1.01**	**1.01**	**1.12**	**1.08**
NN4B 2006–2012				
Female, all live, singleton births				
VLBW	**0.97**	**0.96**	**0.91**	**0.88 (0.86 to 0.91)**
LBW	**0.98**	**0.98**	**0.94**	**0.94**
HBW	**1.01**	**1.01**	**1.03**	**1.02**
Term (≥37 weeks’ gestation), n=2 172 435				
VLBW	0.97 (0.93 to 1.02)	0.96 (0.92 to 1.01)	0.91 (0.80 to 1.06)	0.88 [0.78 to 1.03]
LBW	**0.98**	**0.98**	**0.94**	**0.94**
HBW	1.00	1.01	1.00	1.03
Preterm (<37 weeks’ gestation), n**=**120 665				
VLBW	**0.98**	**0.98**	**0.94 (0.91 to** 0.97)	**0.94 (0.91 to 0.97)**
LBW	**0.98**	0.99	**0.94**	0.97
HBW	1.03 (0.99 to 1.07)	1.03 (0.99 to 1.06)	1.09 (0.97 to 1.23)	1.09 (0.97 to 1.19)
Male, all live, singleton births				
VLBW	**0.97**	**0.97**	**0.91**	**0.91**
LBW	**0.98**	**0.98**	**0.94**	**0.94**
HBW	**1.01**	**1.01**	**1.03**	**1.03**
Male term (≥37 weeks’ gestation) n=2 270 840				
VLBW	0.97 (0.93 to 1.02)	0.97 (0.92 to 1.02)	0.8 (0.8 to 1.06)	0.91 (0.78 to 1.06)
LBW	**0.98**	**0.98**	**0.94**	**0.94**
HBW	**1.01**	**1.01**	**1**	**1.03**
Male preterm (<37 weeks’ gestation), n=144 829				
VLBW	**0.98**	**0.98**	**0.94 (0.91 to** 0.97)	**0.94**
LBW	**0.98**	**0.99**	**0.94**	**0.97**
HBW	1.00 (0.97 to 1.03)	1.00 (0.98 to 1.03)	1.00 (0.91 to 1.09)	1.00 (0.94 to 1.09)

*OR CIs not presented when these were ≤0.03 g or less wide; significant results in bold.

†Adjusted for maternal age, marital status, area-level deprivation, area-level ethnicity (ONS) and individual ethnicity (NN4B).

‡Birth weight change/OR pooled for whole study period ONS (1986–2012), NN4B (2006–2012).

HBW, high birth weight; LBW, low birth weight; NN4B, NHS Numbers for Babies; ONS, Office for National Statistics; VLBW, very low birth weight.

## Discussion

We observed a ~40 g increase in the average birth weight of live, singleton births in England and Wales between 1986 and 2012, mainly driven by increases in the late 1980s and 2000s. During the same period the risk of a low birth weight decreased and the risk of a high birth weight increased. Data for the most recent period (2006–2012) with information on gestational age showed that the increase in the average birth weight was greater in preterm births and there was a slight decrease in the risk of being born preterm over the same time period.

The increase in birth weight found in this study (1986–2012) is consistent with trends from the early 1970s to the mid-1990s in Norway,^17^ Sweden,^18^ Denmark,^19^ France^20^ and North America.^30 31^ All these countries saw an increase in mean birth weight, with some also reporting an increase in high birth weight births. Our observed increase of 43–44 g (1986–2012) is consistent with the USA (1978–1996: 57 g),^30^ Canada (1981–1997: 35 g)^31^ and Sweden (1992–2001: 35 g),^18^ but smaller than that seen in Denmark (1973–2003: 160 g)^19^ and Norway^17^(1967–1998: 100 g), although the periods are not identical. Explanations for these birth weight shifts included concurrent changes in maternal characteristics and behaviour, with smoking, maternal age and BMI having the greatest impact.^18 19 30^

This study uses >98% of all live, singleton records in England and Wales and spans nearly three decades. Excluded births had a lower mean birth weight, a higher area-level percentage of non-white ethnicity and greater area-level deprivation, which may have led to an overestimate of the birth weight increase. There was evidence that the risk of being born preterm was reduced between 2006 and 2012 (online supplementary table 3), which would decrease the risk of being born with a low birth weight; however, a major limitation of ONS births is the lack of gestational age, which makes it impossible to say whether the increase in birth weight is due to an increase in fetal growth or length of gestation. Analysis of NN4B data with gestational age from 2006 onwards suggested a greater increase in birth weight in preterm births. This is inconsistent with birth register studies from North America^5 31^ and Norway^17^ in the 1980s and 1990s, which found an increase in birth weight among term births, but a decrease in birth weight in preterm. This was attributed to an increase in induced births and the use of caesarean sections among preterm births. Without gestational age prior to 2006, we cannot say whether births during the 1980s and 1990s had a similar divergence in birth weight between preterm and term births.

Decreases in maternal smoking have been linked to increases in birth weight in Denmark^19^ and Canada^30^ during the 1980s and 1990s. According to ONS the proportion of women aged 16–49 in Great Britain who smoke fell from 36% in 1986 to 25% in 2012.^32^ The Health and Social Care Information Centre Infant Feeding Survey estimated that the proportion of UK mothers who smoked during pregnancy fell from 33% in 2005 to 26% in 2010.^33^ Babies whose mothers smoke have an average birth weight 150 g lower than those whose mothers do not.^8^ In 2007 the average birth weight in England and Wales began to increase after a 16-year period of no change, which corresponds to the year of the introduction of smoke-free legislation in England (July 2007) and Wales (April 2007). Previous short-term analyses have found this legislation to be associated with reductions in very low birth weight, low birth weight and preterm births and a 19 g increase in mean term birth weight 5 months after implementation.^34^

Younger mothers (<30 years) had lighter babies and showed less increase in birth weight compared with older mothers (≥30 years). Between 1986 and 2012 there was a marked shift in maternal age away from younger mothers (<20 years) (online supplementary figure 3), and by 2012 nearly half of all mothers were aged 30 or older. Overall older mothers tend to weigh more, be less deprived and to smoke less, all factors that can increase birth weight.^12^ However, maternal age was not the strongest predictor of birth weight; its relative importance in the regression model was ~15% for both sexes. This suggests that increasing maternal age may not be the underlying cause of the birth weight trends observed.

Ethnicity and deprivation both had the largest relative importance in the regression model (~35%). The mean percentage of area-level non-white ethnicity increased from 10.7% to 12.0% from 1986 to 2012. Data on individual-level ethnicity were only available from 2006 onwards and area-level information from the census prior to this. Adjusting for individual-level rather than area-level ethnicity in NN4B increased the observed annual increase in birth weight (online supplementary table 5), suggesting that adjustments for area-level ethnicity may have led to an underestimate in birth weight increases. Additionally adjusting for the more ethnic groups (percentage Asian or black) at COA did not change the observed effect size (online supplementary table 6). There were limited individual socioeconomic data available on a 10% sample of the data and covering changes in the classifications used over the study period. Therefore only the census area-level data were used to adjust for deprivation, possibly leading to some residual confounding. However there is evidence that area-based socioeconomic measures are better discriminators of birth weight than individual social class status,^35^ so it is unlikely that individual socioeconomic data would have substantially changed our findings.

To determine the concurrent effects of maternal obesity, ethnicity and socioeconomic status on birth weight, different data sources would need to be used, for example nationally representative cohort studies or linked routine birth data sets.^25^

The rise in being overweight or obese is likely to have influenced birth weight as an increasing maternal BMI has been strongly linked with increased birth weight and risk of a high birth weight.^36^ An analysis of 34 maternity units in England with >619 000 births between 1989 and 2007 found the prevalence of maternal obesity in the first trimester had doubled, from 8% in 1989 to 16% in 2007.^37^ There is also evidence that black and South Asian mothers have a higher incidence of maternal obesity as compared with white mothers,^38^ and there has been a concurrent increase in non-white ethnicity in England and Wales. It is a limitation of our analysis that the data do not have information on maternal height or weight.

Other potential contributory factors to the increase in birth weight are a decrease in the levels of ambient air pollution and changes in antenatal care. UK surveillance data show a decline of more than 60% in the emission of particulate matter PM_2.5_, PM_10_ and nitrogen oxides between 1986 and 2012.^39^ A study consisting of 14 mother–child cohorts from 12 different European countries found that exposure to nitrogen dioxide and PM_2.5_ and PM_10_ during pregnancy is associated with restricted fetal growth, and a higher risk of a low birth weight.^40^ A recent study from Beijing found that the short-term but substantial decrease in air pollution during the 2008 Summer Olympics was associated with a transient increase in birth weight.^41^ There is evidence that appropriate antenatal care can prevent low birth weight,^7^ and improvements in antenatal care in England and Wales over the study period might explain some of the observed decrease in low birth weight.

The reduction seen in the proportion of births that are low birth weight is a beneficial trend as low birth weight is a major risk factor for infant mortality and infant mortality. Rates for the same time period have decreased from 9.6 deaths per 1000 live births in 1986 to 4.0 per 1000 live births in 2012.^42^ However, the concurrent increase in the proportion of high birth weight births may result in increased risks of infant morbidity, obstetric complications and future risks such as adult obesity.^4–6^

## Conclusion

This study found an increase in mean birth weight among live, singleton births in England and Wales between 1986 and 2012. The concurrent decrease in the risk of low birth weight births and the increase in the risk high birth weight births suggest that overall the birth weight distribution of live, singleton births has shifted towards heavier births. Some of the observed increase is explainable by increases in maternal age, reductions in smoking and the introduction of smoke-free legislation, and increases in maternal obesity. Given the recognised strong association between birth weight and disease in both infancy and adulthood, improved understanding of the trends and determinants of birth weight in England and Wales is important to plan, implement and evaluate birth weight interventions.
