# Supplementary material for: Birth weight trends in England and Wales (1986–2012): babies are getting heavier
Source: Arch Dis Child Fetal Neonatal Ed. 2017 Aug 5;103(3):F264–70. doi: 10.1136/archdischild-2016-311790 (PMC5916100; doi:10.1136/archdischild-2016-311790)
Supplement: Supplementary file 1 [file fetalneonatal-2016-311790supp001.docx]

**Supplemental material:**

**Supplemental Figure 1: Flowcharts of births included and excluded in analyses 1986 to 2012.**

**1a: ONS Births**

17,502,059

Live, singleton births 1986-2012 extracted

247,435

Births excluded due to missing data (n = 236,381) or unreliable observations (n =11,054)

17,254,624

Live, singleton births 1986-2012 analysed

**1b: NN4B**

4,786,396

Live, singleton births 2006-2012 extracted

Births excluded due to missing data or unreliable observations (n =77,627)

4,708,769

Live, singleton births 2006-2012 analysed

**Supplemental table** 1**: ONS births characteristics of included and excluded in analysis (1986 and 2012)**

| Characteristics | Included  (n = 17,254,624) | Excluded  (n = 247,435) | P-value* |
| --- | --- | --- | --- |
| Female (%) | 48.7 | 48.6 | 0.3 |
| Mean birth weight (g) | 3354.9 | 3316.6 | <0.001 |
| Maternal age group (%) |  |  | <0.001 |
| <20 | 7.1 | 6.9 |  |
| 20-24 | 21.3 | 21.9 |  |
| 25-29 | 30.5 | 32.1 |  |
| 30-34 | 26.6 | 25.7 |  |
| 35-39 | 12.1 | 11.1 |  |
| ≥40 | 2.5 | 2.3 |  |
| Marital status (%) |  |  | <0.001 |
| Married. | 62.7 | 66.0 |  |
| Not married, single registration. | 7.2 | 8.1 |  |
| Not married, co-registration, parents co-habiting. | 22.7 | 18.4 |  |
| Not married, co-registration, parents living on different address. | 7.5 | 7.5 |  |
| Carstairs 2001 quintile (%) |  |  | <0.001 |
| 1 | 17.3 | 14.4 |  |
| 2 | 18.0 | 16.0 |  |
| 3 | 19.2 | 18.7 |  |
| 4 | 20.2 | 21.0 |  |
| 5 | 25.3 | 29.9 |  |
| Mean non-white area-level ethnicity (%) | 11.3 | 15.5 | <0.001 |

**Test for difference performed using chi-square for categorical variables and t-test for continuous variables.*

**Supplemental table 2: NN4B characteristics of included and excluded in analysis (2006 and 2012)**

| Characteristics | Included  (n = 4,708,769) | Excluded  (n = 77,627) | P-value* |
| --- | --- | --- | --- |
| Female (%) | 48.7 | 48.5 | 0.2 |
| Mean birthweight (g) | 3371.4 | 3234.7 | <0.001 |
| Maternal age group (%) |  |  | <0.001 |
| <20 | 5.9 | 7.4 |  |
| 20-24 | 19.1 | 21.5 |  |
| 25-29 | 27.3 | 27.4 |  |
| 30-34 | 28.0 | 25.7 |  |
| 35-39 | 16.0 | 14.4 |  |
| ≥40 | 3.7 | 3.6 |  |
| Marital status (%) |  |  | <0.001 |
| Married. | 54.0 | 49.9 |  |
| Not married, single registration. | 6.2 | 8.0 |  |
| Not married, co-registration, parents co-habiting. | 30.2 | 30.12 |  |
| Not married, co-registration, parents living on different address. | 9.7 | 12.0 |  |
| Carstairs 2001 quintile (%) |  |  | <0.001 |
| 1 | 16.5 | 13.9 |  |
| 2 | 17.2 | 14.4 |  |
| 3 | 19.5 | 17.7 |  |
| 4 | 21.2 | 20.3 |  |
| 5 | 25.6 | 33.7 |  |
| Mean non-white area-level ethnicity (%) | 12.0 | 12.7 | <0.001 |
| Individual ethnicity |  |  | <0.001 |
| White | 77.0 | 74.9 |  |
| Black | 5.4 | 6.2 |  |
| Asian | 10.2 | 11.0 |  |
| Other | 7.3 | 7.8 |  |
| Mean gestational age (weeks) | 39.3 | 38.0 | <0.001 |

**Test for difference performed using chi-square for categorical variables and t-test for continuous variables.*

**Supplemental table 3: Risks of being born pre-term in all, live singleton births 2006-2012 (NN4B data)**

|  | **Unadjusted yearly Odds Ratio [95% CI]** | **Adjusted^1^ yearly Odds Ratio [95% CI]** |
| --- | --- | --- |
| **NN4B 2006-2012 – All live, singleton births - Pre-term** | | |
| Female | **0.98 [0.98;0.98]** | **0.98 [0.97;0.98]** |
| Male | **0.98 [0.98;0.98]** | **0.97 [0.98;0.98]** |

^1^Adjusted for maternal age, marital status, area-level deprivation, individual ethnicity

**Supplemental table 4: Temporal trends in mean birthweight (grams) in all births 1986**- **2012 (ONS data) by maternal age**

| **Maternal age** | **Unadjusted yearly birthweight change [95% CI]** | **Adjusted^1^ yearly birthweight change [95% CI]** | **Unadjusted birthweight change [95% CI] 1986-2012** | **Adjusted^3^ birthweight change [95% CI] 1986-2012** |
| --- | --- | --- | --- | --- |
| **Male** | | | | |
| <30 years | 0.1 [0.0-0.2] | 1.2 [1.1-1.2] | 0.6 [0.2-1.1] | 8.1 [7.7-8.6] |
| ≥30 years | 2.4 [2.3-2.4] | 2.5 [2.4-2.6] | 16.5 [16.0-17.0] | 17.3 [16.9-17.9] |
| **Female** | | | | |
| <30 years | 0.1 [0.1-0.2] | 1.3 [1.2-1.3] | 1.0 [0.6-1.4] | 8.9 [8.5-9.3] |
| ≥30 years | 2.4 [2.3-2.4] | 2.5 [2.4-2.6] | 16.5 [16.0-17.0] | 17.4 [16.9-17.9] |

^1^ Adjusted for marital status, area-level deprivation and area-level ethnicity

**Supplemental Figure 2: Temporal trends in proportions of live, singleton births in England and Wales (1986-2012) with a very low birth weight (VLBW), low birth weight (LBW), and high birth weight (HBW).**

**Supplemental figure 3: a) Proportion of births (%) by maternal age group, live, singleton births in England and Wales (1986 and 2012)^1^ b) Mean birthweight by maternal age and sex** ^1^ imputed data in 1991

A

**
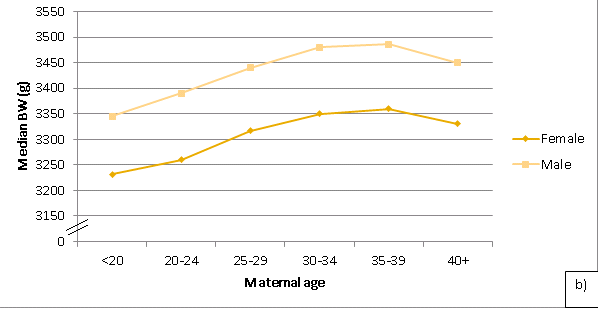
**

B

**Supplemental table 5: Sensitivity analysis - Annual trends in mean birthweight (grams) adjusting for area or individual level ethnicity (2006**-**2012)**

|  | **Unadjusted yearly change [95% CI]** | **Adjusted^1^ yearly change [95% CI] – Area-level ethnicity** | **Adjusted^2^ yearly change [95% CI] – individual ethnicity^2^** |
| --- | --- | --- | --- |
| **Male** | | | |
| birthweight (grams) | **3.8 [3.4:4.1]** | **3.7 [3.3;4.0]** | **4.4 [4.0;4.8]** |
| **Term (≥37 weeks gestation)** | | | |
| birthweight (grams) | **2.5 [2.2;2.8]** | **2.3 [2.0;2.6]** | **3.0 [2.7;3.3]** |
| **Pre-Term (<37 weeks gestation)** | | | |
| birthweight (grams) | **4.4 [2.3;6.2]** | **4.2 [2.4;5.9]** | **4.3 [2.5;6.1]** |
| **Female** | | | |
| birthweight (grams) | **3.4 [3.0;3.7]** | **3.4 [3.0;3.7]** | **3.9 [3.6;4.3]** |
| **Term (≥37 weeks gestation)** | | | |
| birthweight (grams) | **2.3 [1.9;2.6]** | **2.2 [1.9;2.5]** | **2.7 [2.4;3.1]** |
| **Pre-Term (<37 weeks gestation)** | | | |
| birthweight (grams) | **4.3 [2.3;6.2]** | **4.2 [2.3;6.1]** | **4.3 [2.4;6.3]** |

^1^Adjusted for maternal age, marital status, area-level deprivation and area-level ethnicity.

^2^Adjusted for maternal age, marital status, area-level deprivation and individual level ethnicity.

**Supplemental table 6: Sensitivity analysis - Annual trends in mean birthweight (grams) adjusting for area-level ethnicity split by ethnic groups (1986**-**2012)**

| **Yearly birthweight change (grams) [95% CI]** | **Unadjusted** | **Adjusted^1^ + percentage white** | **Adjusted^1^ + percentage black** | **Adjusted^1^ + percentage Asian** | **Adjusted^1^ + percentage white, black & Asian** |
| --- | --- | --- | --- | --- | --- |
| **ONS births 1986-2012** | | | | | |
| **Female births** | **1.4 [1.3;1.4]** | **1.6 [1.5;1.6]** | **1.4 [1.3;1.4]** | **1.6 [1.5;1.6]** | **1.6 [1.6;1.7]** |
| **Male births** | **1.4 [1.4;1.5]** | **1.6 [1.6;1.7]** | **1.4 [1.3;1.4]** | **1.6 [1.6;1.7]** | **1.6 [1.6;1.7]** |

^1^Adjusted for maternal age, marital status, area-level deprivation

**Supplemental table 7: Mean annual birthweight of all live, singleton births in England and Wales (1986-2012) with years of change – data for figure 2**

| **Year** | **Mean male birthweight (grams)** | **Mean female birthweight (grams)** |
| --- | --- | --- |
| 1986 | 3376 | 3258 |
| 1987 | 3385 | 3267 |
| **1988** | **3399** | **3283** |
| 1989 | 3400 | 3282 |
| **1990** | **3402** | **3283** |
| 1991 | 3406 | 3287 |
| 1992 | 3418 | 3297 |
| 1993 | 3420 | 3303 |
| 1994 | 3419 | 3300 |
| 1995 | 3407 | 3289 |
| 1996 | 3413 | 3291 |
| 1997 | 3410 | 3291 |
| 1998 | 3412 | 3294 |
| 1999 | 3409 | 3292 |
| 2000 | 3416 | 3298 |
| 2001 | 3411 | 3294 |
| 2002 | 3407 | 3291 |
| 2003 | 3406 | 3289 |
| 2004 | 3409 | 3294 |
| 2005 | 3411 | 3296 |
| 2006 | 3412 | 3294 |
| **2007** | **3421** | **3303** |
| 2008 | 3428 | 3309 |
| **2009^1^** | 3428 | **3309** |
| 2010 | 3434 | 3316 |
| 2011 | 3435 | 3315 |
| 2012 | 3436 | 3316 |

**Bold =** Potential temporal points of change in the average yearly birthweight

^1^ Potential points of change in female births only
